# Pharmacomicrobiomics: From Host–Microbiome–Drug Interactions to Clinical Translation in Precision Medicine

**DOI:** 10.3390/metabo16090602

**Published:** 2026-08-23

**Authors:** Guilherme Araújo, Sara Domingues, Gabriela Jorge da Silva, Tiago Lima

**Affiliations:** 1 Faculty of Pharmacy, University of Coimbra, 3000-458 Coimbra, Portugalsaradomingues@ff.uc.pt (S.D.); 2 CNC-UC-Center for Neuroscience and Cell Biology, University of Coimbra, 3004-517 Coimbra, Portugal; 3 CiBB-Centre for Innovative Biomedicine and Biotechnology, University of Coimbra, 3004-548 Coimbra, Portugal; 4 Department of Medical and Health Sciences, School of Health and Human Development, University of Évora, 7004-516 Évora, Portugal; 5 Comprehensive Health Research Centre (CHRC), University of Évora, 7004-516 Évora, Portugal

**Keywords:** pharmacomicrobiomics, gut microbiota, microbial biotransformation, pharmacokinetics, pharmacodynamics, therapeutic response

## Abstract

Marked interindividual variability in drug response remains a major challenge in clinical pharmacology and cannot be fully explained by host genetics alone. Increasing evidence indicates that the gut microbiota constitutes an additional determinant of drug efficacy and toxicity through bidirectional interactions with pharmacological therapies. This recognition has led to the emergence of pharmacomicrobiomics, a field that investigates how microbial communities influence drug disposition and response, and how drugs, in turn, alter the microbiome. Microbiome-mediated effects on drug efficacy and toxicity have been described across several therapeutic areas, including oncology, multiple sclerosis, and type 2 diabetes mellitus. Although these findings are promising, most mechanistic evidence derives from preclinical and animal studies, with relatively limited validation in controlled clinical trials. Strategies to modulate the gut microbiota, including prebiotics, probiotics, and faecal microbiota transplantation, have shown preliminary promise in optimising drug efficacy and reducing adverse effects, although methodological heterogeneity and incomplete mechanistic understanding limit their current clinical application. The identification of robust microbiome-derived biomarkers and the integration of multi-omics approaches, particularly metabolomics, are expected to accelerate the translation of pharmacomicrobiomics into precision medicine. This review summarises current evidence regarding microbiome–drug interactions, the mechanisms underlying microbiome-mediated modulation of pharmacokinetics and pharmacodynamics, emerging therapeutic strategies, and the challenges that remain before pharmacomicrobiomics can be implemented in clinical practice.

## 1. Introduction

Pharmacomicrobiomics is an inherently multidisciplinary field at the interface of pharmacology, microbiology and systems biology, concerned with understanding how the gut microbiome modulates drug disposition and therapeutic response [1]. Growing evidence indicates that interindividual variability in drug efficacy and toxicity cannot be fully explained by host genetics alone, and that microbial contributions to pharmacokinetics and pharmacodynamics may represent a substantial, and previously underappreciated, source of variability [2,3].

The conceptual foundations of pharmacomicrobiomics date back almost a century. As early as 1937, intestinal microorganisms were shown to mediate the biotransformation of prontosil into its active sulfonamide form, thereby demonstrating that microbial metabolism could be essential for drug efficacy. Since then, numerous studies have suggested that host–drug–microbiome interactions are fundamentally bidirectional [2,3,4]. On the one hand, pharmaceutical compounds can alter the composition and functional capacity of the gut microbiota; on the other, microbial enzymatic activity can profoundly influence drug bioavailability, pharmacological activity and toxicity. These reciprocal interactions are now recognised as relevant across a wide range of therapeutic classes [5,6,7].

The gut microbiome has therefore been increasingly conceptualised as a highly dynamic metabolic organ, characterised by remarkable plasticity and responsiveness to environmental cues [8]. From a therapeutic perspective, this plasticity renders the microbiome an attractive target for intervention. Strategies such as dietary modulation, prebiotics, probiotics and faecal microbiota transplantation (FMT) have been proposed as means to enhance drug efficacy or to mitigate adverse effects, although their clinical integration remains uneven and context-dependent [9]. Collectively, these developments suggest that pharmacomicrobiomics may contribute to a more refined and mechanistically grounded model of precision pharmacotherapy.

The present review examines how the gut microbiome influences therapeutic response to pharmacological interventions. Specifically, it synthesises current knowledge on (i) host–microbiome–drug interactions, with emphasis on microbial contributions to drug metabolism; (ii) the impact of microbiome variability on pharmacodynamic effects and clinical outcomes in selected disease areas, including oncology, metabolic disorders and immune-mediated diseases; and (iii) emerging strategies for microbiome modulation aimed at improving treatment efficacy and safety. Finally, key methodological, translational and regulatory challenges are discussed, highlighting areas where evidence remains fragmentary or contradictory.

## 2. Human Microbiome: Definition, Functions, and Modulators

The gut microbiome is increasingly conceptualised as a metabolic organ in its own right. The bacterial component alone encodes over three million genes, roughly 150-fold more than the human genome, and the metabolic reactions it mediates are not simply redundant with those of the host but complement and extend them in ways that directly affect drug disposition [8]. Dominated by two phyla, *Bacillota* and *Bacteroidota*, with minor contributions from *Actinomycetota*, *Verrucomicrobiota*, and others, the gut microbiota nonetheless exhibits substantial interindividual variability in taxonomic composition. An individual’s microbial profile can be almost as distinctive as a fingerprint [10,11]. Critically for pharmacomicrobiomics, this taxonomic diversity does not translate into equivalent functional diversity. Up to 82% of metabolic pathways can be shared between microbiomes with almost no species in common [10,12]. This conservation of function across variable taxonomic compositions is precisely what makes microbial contributions to drug metabolism both widespread and difficult to predict from composition data alone.

Among the metabolically relevant outputs of the gut microbiota, short-chain fatty acids (SCFAs), primarily acetate, propionate, and butyrate, and secondary bile acids, generated from primary bile acids through bacterial 7α-dehydroxylase activity, are of particular relevance to pharmacokinetics. Both classes of metabolites modulate the expression and activity of membrane drug transporters and drug-metabolising enzymes, as discussed in Section 3 [12,13,14,15,16]. Perturbations in the microbial community, whether driven by antibiotic exposure, dietary changes, or other environmental factors, alter the production of these metabolites and can consequently shift the pharmacokinetic profile of co-administered drugs [10,17,18].

The composition and functional capacity of the gut microbiota are shaped by a dynamic interplay of factors including age, mode of delivery, diet, antibiotic use, psychological stress, and host genetics. Together, these factors contribute to the marked interindividual variability in gut microbiota composition documented in the literature [10,19]. Of these factors, antibiotic exposure and diet are the most pharmacologically consequential. Antibiotics can profoundly and durably reduce microbial diversity and disrupt the functional repertoire of the gut community [18,20,21,22], while dietary patterns rapidly modulate the abundance of SCFA- and bile acid-producing taxa, with measurable consequences for microbial metabolite output [12,19,20,23]. This variability in microbiota composition and function provides the biological basis for heterogeneity in drug response among individuals receiving the same pharmacological treatment. This biological variability underpins the central question addressed by pharmacomicrobiomics.

## 3. Microbiome–Drug Interactions: Metabolic and Molecular Mechanisms

The gut microbiome exerts both direct and indirect effects on the metabolism of drugs and xenobiotics, influencing therapeutic efficacy as well as treatment safety. These interactions affect key pharmacokinetic and pharmacodynamic processes, including drug absorption, distribution, metabolism, and excretion. As a result, the microbiota may alter how the organism responds to treatment by modifying the bioavailability of drugs and promoting the formation of either active or toxic metabolites, which can ultimately impact therapeutic outcomes and the risk of adverse effects [2,3].

The gut microbiome is often described as a metabolic organ, given its ability for bidirectional interaction with a wide range of drugs. Certain drugs, particularly antibiotics with activity against Gram-positive and anaerobic bacteria, can profoundly alter microbial composition and diversity. Some evidence suggests that early-life antibiotic exposure may interfere with microbiome ontogeny, referring to its normal development and maturation, potentially leading to long-term metabolic, immune, and neurological dysfunctions. Conversely, the gut microbiota can metabolise certain drugs before their absorption and entry into systemic circulation, thereby affecting their relative oral bioavailability, namely the effective amount of drug reaching its intended site of action. This early transformation may therefore play a decisive role in determining therapeutic efficacy [5,6,7].

The marked interindividual variability in microbiota composition, shaped by multiple factors discussed previously, may help explain why clinically similar individuals can exhibit significantly different responses to the same pharmacological treatment [7]. It is therefore becoming increasingly evident that microbiome profiling may represent an important consideration in the development of therapeutic strategies. A deeper understanding of these interactions could support the design of more effective and safer, and potentially personalised, interventions [24,25].

### 3.1. Influence of the Gut Microbiota on the Pharmacokinetics of Oral Drugs

Pharmacokinetics allows the understanding of how the organism influences drug disposition and concentration over time [26]. These parameters determine the onset, duration, and intensity of the therapeutic effect, and are subject to multiple individual factors [24].

The gut microbiota has a significant impact on the pharmacokinetic parameters of orally administered drugs, directly influencing absorption, distribution, and systemic exposure [7]. In particular, it may directly affect oral bioavailability through modulation of membrane transporters, such as P-glycoprotein (P-gp/MDR1), multidrug resistance-associated protein 2 (MRP2), and breast cancer resistance protein (BCRP). These transporters belong to the ATP-binding cassette (ABC) family and regulate intracellular drug retention and transport into the intestinal lumen or bile, acting as efflux pumps [13,14].

Microbial metabolites, including SCFAs produced by bacterial species such as *Faecalibacterium prausnitzii*, *Roseburia* spp., and *Eubacterium rectale*, as well as secondary bile acids such as deoxycholic and lithocholic acids, derived from bacteria with 7α-dehydroxylase activity such as *Clostridium scindens*, have been associated with the regulation of signalling pathways that control the expression of these transporters [13,14,15,16].

**Figure 1:**
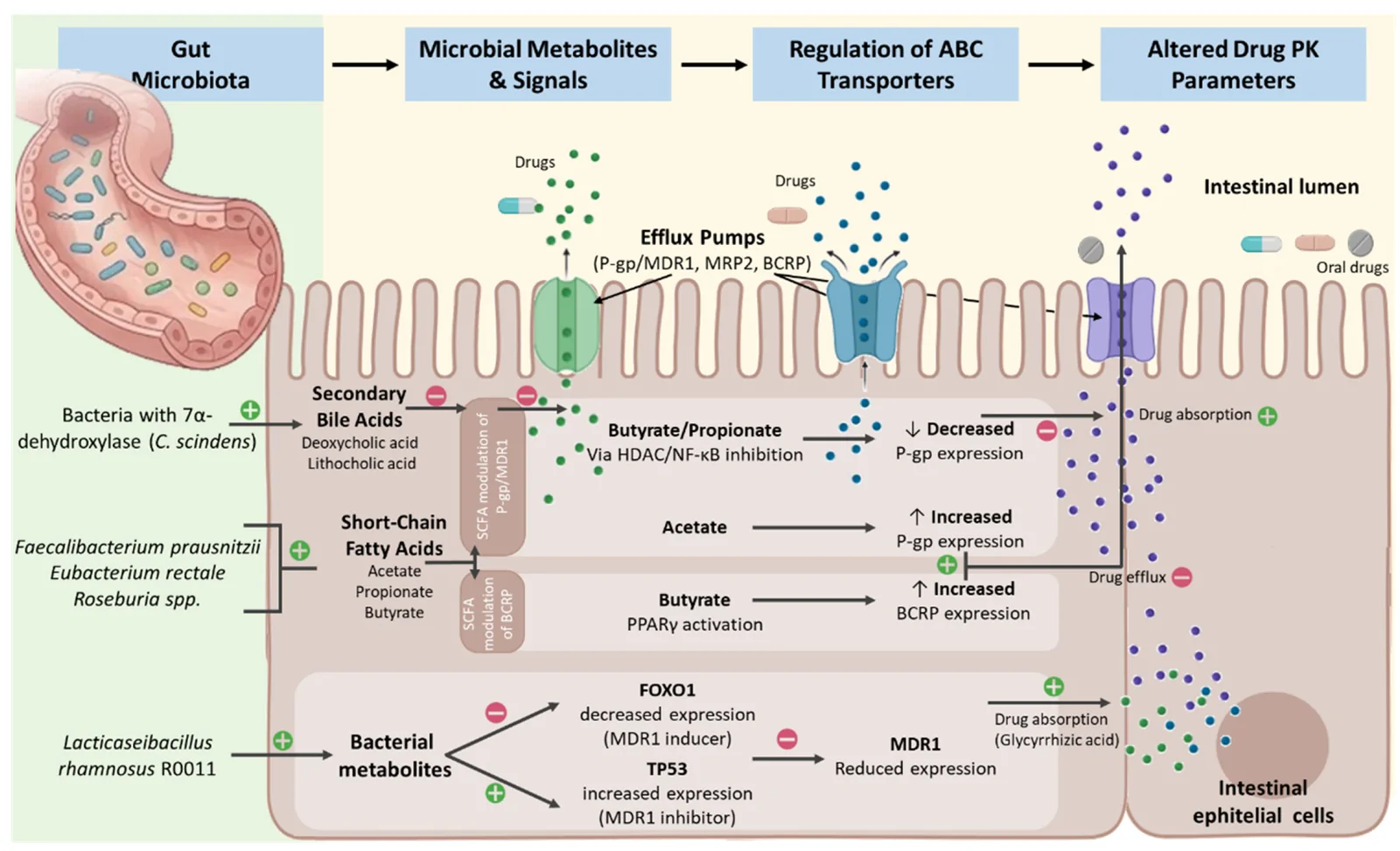
Mechanistic Overview of Gut Microbiota Influence on Oral Drug Pharmacokinetics. The diagram illustrates how specific bacterial species and their microbial metabolites regulate the expression and function of ABC transporters (efflux pumps) within intestinal epithelial cells. Key signalling pathways are shown: butyrate and propionate decrease P-gp expression via HDAC/NF-κB inhibition, whereas acetate increases it. Concurrently, butyrate enhances BCRP expression through PPARγ activation. Downregulation of MDR1 by *Lacticaseibacillus rhamnosus* R0011 metabolites is mediated by decreasing FOXO1 (an inducer) and increasing TP53 (an inhibitor). Secondary bile acids, derived from bacterial 7α-dehydroxylase activity, directly inhibit P-gp-mediated efflux. Ultimately, these microbiota-driven alterations modulate the balance between drug absorption and efflux, directly impacting the systemic bioavailability of orally administered therapeutics (e.g., glycyrrhizic acid).

Butyrate and propionate have been shown to reduce P-gp expression and function, partly through inhibition of the HDAC/NF-κB pathway, which may result in increased intracellular drug retention. In contrast, acetate appears to be associated with increased expression of this transporter [14]. In addition, butyrate may enhance BCRP expression through activation of peroxisome proliferator-activated receptor gamma (PPARγ) [14], as shown in Figure 1.

Studies involving *Lacticaseibacillus rhamnosus* R0011 have demonstrated a significant reduction in MDR1 expression in the ileum. This effect appears to be mediated by microbial metabolites which, although not directly affecting MDR1 function, influence its transcriptional regulation, namely through decreased expression of FOXO1 (an MDR1 inducer) and increased expression of TP53 (an MDR1 inhibitor). The overall effect is reduced MDR1 expression, which may favour the absorption of drugs such as glycyrrhizic acid [14], as illustrated in Figure 1.

Certain bile acids, particularly secondary bile acid derivatives such as taurolithocholate, as well as other bile salts including glycochenodeoxycholate, may inhibit P-gp-mediated drug efflux, either by altering the microenvironment surrounding the transporter or through direct interaction [13]. In addition, it has been proposed that primary bile acids may compete with simvastatin for the same hepatic and intestinal transporters, including MDR1 and MRP2, potentially reducing drug absorption. However, microbial conversion of primary into secondary bile acids may reduce this competition and improve bioavailability of the drug [13,14].

A related example is the administration of lovastatin in formulations containing sodium deoxycholate. This secondary bile salt is able to inhibit the action of P-glycoprotein, contributing to an increase in the bioavailability of lovastatin. This effect has not been observed with primary bile salts such as sodium glycocholate [13].

The physicochemical intestinal environment, including luminal pH, viscosity, and epithelial barrier integrity, may also be modulated by the microbiota [25,27], thereby influencing drug solubility and the duration of contact with the intestinal mucosa [13]. For instance, the absorption of gliclazide, a sulfonylurea antidiabetic drug, is affected by intestinal pH. Probiotic mixtures containing *Lactobacillus acidophilus*, *Lacticaseibacillus rhamnosus*, and *Bifidobacterium lactis* have been shown to increase its permeability, likely through SCFA-mediated acidification of the intestinal environment, which favours the non-ionised form of the drug and enhances absorption [3,27].

Absorption and efficacy of levodopa, widely used in the treatment of Parkinson’s disease, may be impaired in the presence of *Helicobacter pylori* infection. This bacterium can alter gastric motility, damage the duodenal mucosa, and increase histidine decarboxylase activity, leading to conversion of levodopa into dopamine (a non-absorbable form) before absorption. Clinical studies have shown that eradication of *Helicobacter pylori* results in improved levodopa bioavailability with consequent clinical improvements in patients [13,27].

The microbiota may also influence intestinal transit time. For example, *Lactobacillus reuteri* KCTC3679 has been shown to reduce paracetamol bioavailability in animal models, an effect attributed to the stimulation of peristalsis and consequent reduction in intestinal transit time, thereby limiting drug absorption [13].

Taken together, these findings highlight the role of the gut microbiota in modulating multiple aspects of pharmacokinetics, particularly drug absorption and bioavailability, and support its contribution to interindividual variability in therapeutic response.

### 3.2. Microbial Mechanisms of Drug Biotransformation

The gut microbiome plays a significant role in drug metabolism, particularly in the conversion of prodrugs into active compounds and in the inactivation of pharmacologically active substances. These processes are mediated by microbial enzymatic pathways that differ from those of the host and are predominantly carried out by anaerobic bacteria in the colon, which represents one of the regions with the highest microbial density and metabolic activity [7,26].

Representative examples of microbiome-mediated drug biotransformation, including the underlying mechanisms and clinical implications, are summarised in Table 1. Beyond conventional pharmaceutical agents, this bidirectional relationship also extends to herbal medicines, exemplified in Table 1 by Daikenchuto (TU-100), a Kampo medicine whose gut bacterial biotransformation into the bioactive ginsenoside metabolite compound K parallels the drug-activation mechanisms detailed in the following subsections.

#### 3.2.1. Activation of Prodrugs

While host cytochrome P450 enzymes represent a major pathway for drug metabolism, microbial metabolism may also be essential for the activation of certain prodrugs into their active forms [7].

One of the earliest and most illustrative examples dates back to the 1930s, when sulphanilamide was identified as the active metabolite of prontosil. Prontosil is administered in an inactive form and subsequently converted into sulphanilamide, a compound with antibacterial activity, through cleavage of an azo bond, a reaction mediated by bacterial azoreductases [3,4].

Another widely studied example is sulfasalazine, a prodrug composed of sulfapyridine and 5-aminosalicylic acid (5-ASA), an anti-inflammatory component, linked by an azo bond [4]. This compound is primarily used in inflammatory bowel disease and is administered in an inactive form. The azo bond remains stable in the entire upper GIT, allowing the intact drug to reach the colon, where it is mainly metabolised by bacterial species, including *Bacteroides* spp., *Clostridium* spp., and *Eubacterium* spp. Cleavage of the azo bond by bacterial azoreductases releases 5-ASA, which is responsible for the local anti-inflammatory effect [3,7,26].

Similarly, olsalazine and loperamide oxide are also considered prodrugs whose activation occurs via reduction reactions mediated by the gut microbiota. In the case of olsalazine, bacterial azoreductases appear to be directly involved in its conversion [4,69], whereas for loperamide oxide, although microbial involvement is well established, the specific enzymes responsible for its activation have not yet been fully identified [4,43].

Metronidazole, an antimicrobial agent, is another prodrug whose activation is also influenced by microbial metabolism. This process involves the reduction of the nitro group to an amine (nitroreduction), particularly in the presence of anaerobic bacteria such as *Clostridium perfringens*. The pharmacological consequences of such reactions depend on the chemical structure of the drug and the stability of intermediate products, and may yield either active compounds or potentially toxic metabolites. In the case of metronidazole, nitroreduction produces the active antimicrobial metabolite [3].

Lovastatin is a lactone prodrug, requiring hydrolysis to its active β-hydroxyacid form to exert its lipid-lowering effect [4]. This reaction may be mediated not only by host enzymes but also by microbial esterases present in the gut [25,26].

#### 3.2.2. Inactivation of Active Drugs

The gut microbiota may also contribute to the inactivation of pharmacologically active compounds through specific enzymatic transformations.

Digoxin, a cardiac glycoside, is reduced to the inactive metabolite dihydrodigoxin by cardiac glycoside reductase, an enzyme expressed by *Eggerthella lenta*. This process is influenced by several factors, including the presence of the *cgr* operon, which encodes proteins responsible for this reduction; dietary components, since the presence of arginine in the intestinal lumen has been shown to inhibit enzyme expression and thereby reduce digoxin inactivation; and interindividual variability in the presence or absence of specific *Eggerthella lenta* strains capable of expressing these enzymes [2].

#### 3.2.3. Reactivation of Metabolites and Microbiota-Induced Toxicity

One of the most extensively studied mechanisms in microbiome-mediated drug metabolism is the deconjugation of glucuronides by bacterial β-glucuronidases. These enzymes, expressed by several intestinal bacteria, particularly within the *Bacillota* and *Bacteroidota* phyla, hydrolyse drug–glucuronide conjugates, leading to reactivation of compounds previously inactivated by the liver and enabling their reabsorption in the intestine [70]. The consequence is increased systemic exposure to the drug, which in some cases may prolong therapeutic effects, whereas in others it may enhance local toxicity [4,70]. For example, increased β-glucuronidase activity in the gut has been associated with enhanced reactivation of morphine, leading to increased bioavailability and prolonged analgesic action [70]. In contrast the reactivation of irinotecan’s glucuronidated metabolite in the colon is strongly associated with gastrointestinal toxicity, as detailed below [27,70].

Non-steroidal anti-inflammatory drugs (NSAIDs), such as diclofenac and indomethacin, also undergo hepatic glucuronidation and are subsequently excreted into the intestine via the enterohepatic circulation. In the intestinal lumen, bacterial β-glucuronidases cleave these conjugates, regenerating the active drug, which can be reabsorbed by the enterocytes. This process has been associated with NSAID-induced enteropathy, including mucosal damage and intestinal inflammation [27].

#### 3.2.4. Indirect Interference in Drug Metabolism

Beyond direct enzymatic transformations, the microbiota may indirectly influence drug metabolism by producing metabolites that compete with host enzymes or by modulating host enzymatic activity. These interactions may affect therapeutic efficacy of the drugs or increase the risk of toxicity.

Tacrolimus, a widely used immunosuppressant in transplant patients, has a narrow therapeutic index, with subtherapeutic concentrations leading to transplanted organ rejection, while supratherapeutic levels are associated with nephrotoxicity and neurotoxicity [27]. Although direct microbial inactivation of the drug has not been demonstrated, one study reported that patients with higher abundance of *Faecalibacterium prausnitzii* required higher doses to achieve therapeutic plasma concentrations [63]. The authors proposed that tacrolimus absorption and metabolism are related to intestinal mucosal integrity, which depends, in part, on butyrate levels produced by species such as *Faecalibacterium prausnitzii*. This mechanism may explain the lower therapeutic efficacy observed in individuals with high abundance of this species [27].

Paracetamol, an analgesic and antipyretic drug, is primarily metabolised in the liver through glucuronidation and sulfation pathways [4]. Although the underlying mechanisms are incompletely understood, interindividual variability in paracetamol metabolism has been associated with microbial metabolites such as p-cresol, which may compete with paracetamol for sulfotransferase enzymes. Elevated baseline p-cresol levels may reduce paracetamol sulfation through competition, potentially shifting its metabolism towards alternative pathways and increasing the risk of hepatotoxicity [4,58].

Another classic example of microbiome-mediated drug–drug interaction involves sorivudine, whose microbial metabolite inhibits dihydropyrimidine dehydrogenase, thereby increasing 5-fluorouracil toxicity. Although no longer clinically relevant, this interaction illustrates how microbial metabolism may indirectly alter host drug-processing pathways [3,71].

The gut microbiota is also capable of carrying out a wide range of further metabolic reactions, including acetylation, deacetylation, decarboxylation, dehydroxylation, demethylation, and dehalogenation. These transformations may significantly alter the chemical structure of certain drugs, contributing both to their pharmacological activity and to the formation of potentially toxic metabolites [3].

### 3.3. Influence of the Gut Microbiota on Pharmacodynamics and Therapeutic Response in Diabetes, Multiple Sclerosis and Oncology

Microbiota modulation is also closely linked to the pathophysiology of several diseases, with particular impact in oncology, and in neurological, metabolic, and infectious conditions [72]. Understanding the interactions between the microbiota and therapeutic agents is therefore essential for developing and refining individualised treatment strategies [25].

#### 3.3.1. Type 2 Diabetes Mellitus

Differences between the gut microbiota of healthy individuals and patients offer an opportunity to characterise normal microbiota and to identify patterns associated with specific diseases. In T2D, intestinal dysbiosis is characterised by a reduction in butyrate-producing bacteria, including *Bifidobacterium* spp., *Akkermansia* spp., and several *Clostridium* species, alongside an increase in other microorganisms such as *Lactobacillus* spp. and potentially pathogenic organisms including *Escherichia coli* [15,73]. Given the prominent role of the gut microbiome in T2D, pharmacomicrobiomic studies have been proposed to explain the interactions between the microbiome and antidiabetic agents.

In the case of metformin, the most widely used oral antidiabetic drug, there is evidence that its administration improves hyperglycemia in T2D patients by increasing the abundance of beneficial bacterial species, which produce SCFAs that contribute to the therapeutic effect by improving insulin sensitivity [74]. In some individuals, however, the gut microbiome may negatively influence drug tolerability. An increase in *Escherichia* spp., associated with virulence factors and fermentation genes, may underlie the gastrointestinal adverse effects commonly observed with metformin, namely intestinal irritation and gas production [75]. Although the mechanisms remain incompletely understood, the microbiota is also thought to modulate the metabolism or absorption of other antidiabetic agents, including SGLT2 and DPP-4 inhibitors [25].

#### 3.3.2. Multiple Sclerosis

The interaction between the gut microbiota and the immune system is particularly relevant in therapeutic contexts that depend on effective immune modulation, such as multiple sclerosis (MS). Disruptions in microbial balance can influence the immune response by altering key cell populations, including regulatory T cells (Treg), which promote immunological tolerance, and the pro-inflammatory Th1 and Th17 cells, which drive the inflammatory pathology associated with MS.

Several studies have identified specific dysbiosis profiles in the gut microbiota of MS patients, suggesting that these alterations may contribute to immune dysregulation and disease progression, as summarised in Table 2. The microbiota therefore emerges as a potential target for therapeutic intervention, not only through its influence on pathophysiology but also through its capacity to modulate treatment efficacy and tolerability [16].

One study evaluating the activity of interferon-β1b, an immunomodulatory therapy used in MS, found that treated patients showed an increase in *Prevotella* spp., a genus associated with beneficial immunoregulatory effects, to levels comparable to those of healthy controls. This shift was accompanied by increased SCFA production, particularly propionate, which carries anti-inflammatory properties mediated through Treg cell induction. These findings suggest that the efficacy of interferon-β1b may be partly dependent on the restoration of a healthy intestinal microbial profile, which in turn supports favourable immune modulation [77].

Conversely, a six-month longitudinal study evaluating dimethyl fumarate (DMF) in MS patients found that treatment induced changes in the bacterial community, including an increase in *Streptococcus* spp. and *Haemophilus* spp. Although DMF is effective in reducing disease activity, these microbial changes were associated with gastrointestinal adverse effects, pointing to the microbiota as a mediator of treatment-related side effects, independent of its role in therapeutic response [78].

#### 3.3.3. Oncology

Irinotecan represents one of the most well-characterised examples of microbiota-mediated modulation of oncological treatment response. Used primarily in colorectal cancer, irinotecan is converted into SN-38, its active metabolite, which is subsequently inactivated by conjugation with glucuronic acid. In the intestine, this conjugation can be reversed by bacterial β-glucuronidase activity, locally reactivating SN-38 and leading to accumulation of the active metabolite, with clinically significant adverse effects including severe diarrhoea [27,70].

Beyond toxicity, the microbiota can also potentiate therapeutic response in oncology, particularly in the context of immunotherapy with immune checkpoint inhibitors such as anti-PD-1 agents. The presence of certain bacterial species, including *Akkermansia muciniphila* and *Enterococcus hirae*, has been associated with improved clinical outcomes in this setting, through dendritic cell activation in lymph nodes, increased IL-12 release, and promotion of CD8+ T-cell and Th1 cell infiltration in the tumour microenvironment [27,79].

Oxaliplatin exerts its antineoplastic effect through induction of DNA damage and generation of reactive oxygen species, and its efficacy can be modulated by the gut microbiota through several mechanisms. Commensal bacteria, including non-enterotoxigenic *Bacteroides fragilis* and members of the *Erysipelotrichaceae* family, stimulate dendritic cells, activate follicular helper T cells, and promote IgG2b antibody production, thereby enhancing CD8+ T-lymphocyte activity. Various microbiota species capable of producing SCFAs such as butyrate can further improve CD8+ T-cell cytotoxic function through histone deacetylase inhibition. *Fusobacterium nucleatum*, frequently found in colorectal cancer contexts, may instead induce chemoresistance to oxaliplatin by activating the TLR4/MYD88 pathway and diverting tumour cells from apoptosis towards autophagy [74,79].

Cyclophosphamide provides a further example of microbiome-dependent therapeutic modulation. This drug can damage the intestinal mucosa, causing villous shortening and increased permeability through impaired tight junction function, allowing translocation of bacteria such as *Enterococcus hirae* and *Lactobacillus johnsonii* into secondary lymphoid organs, namely lymph nodes and spleen. This translocation promotes the differentiation of “pathogenic” Th17 cells and memory Th1 cells, both essential for an effective antitumour immune response. The presence of *Enterococcus hirae* is additionally associated with an increased ratio of intratumoural CD8+ T cells relative to regulatory T cells, potentially reversing chemoresistance [79]. At the same time, cyclophosphamide induces intestinal dysbiosis, with reduced microbial diversity and overgrowth of pathogenic species such as *Escherichia coli* and *Pseudomonas aeruginosa* [24,74]. These alterations are associated with inflammatory response activation and intestinal barrier dysfunction, contributing to increased treatment toxicity [80].

The extent to which these interactions can be deliberately modulated to improve therapeutic outcomes forms the basis of the microbiota-based strategies discussed in the following section.

## 4. Therapeutic Modulation of the Gut Microbiota: Implications for Pharmacomicrobiomics

A growing understanding of the interactions between the gut microbiome, the host, and drugs has driven the development of therapeutic strategies aimed at modulating the intestinal microbiota. Among the most actively studied approaches are prebiotics, probiotics, and FMT. Although these interventions were initially developed to restore eubiosis and treat dysbiosis-associated diseases, accumulating evidence suggests that they can also modify drug pharmacokinetics, microbial metabolism, and consequently the efficacy and safety of various medications. Targeted manipulation of the microbiota therefore represents a promising strategy for the personalisation of pharmacotherapy [9].

### 4.1. Prebiotics

Prebiotics are non-digestible substrates selectively utilised by intestinal microorganisms, conferring benefits to the host through modulation of the gut microbiota [81,82,83]. Among the most studied compounds are inulin, fructooligosaccharides, galactooligosaccharides, and xylooligosaccharides, which preferentially stimulate the growth of genera considered beneficial, including *Bifidobacterium* spp. and *Lactobacillus* spp. [9,84].

Their effects are largely mediated by increased SCFA production, which contributes to the maintenance of intestinal barrier integrity and to the regulation of various metabolic and immunological pathways [85]. In the context of pharmacomicrobiomics, these changes may modify pharmacological response by influencing both the microbiota and the intestinal environment.

Preclinical studies have shown that prebiotics such as inulin and certain oligosaccharides can potentiate the antitumour activity of drugs including 5-fluorouracil and cyclophosphamide [79]. Conversely, other compounds, namely pectin and oat-derived β-glucans, have demonstrated the capacity to reduce methotrexate-induced enterocolitis [79]. Not all effects are beneficial, and some oligosaccharides appear to increase bacterial β-glucuronidase activity, favouring reactivation of irinotecan’s SN-38 metabolite and worsening the gastrointestinal toxicity associated with its administration [79].

These findings demonstrate that prebiotics can influence drug efficacy and toxicity through functional modulation of the gut microbiota, although the mechanisms involved remain only partially understood [79,86].

### 4.2. Probiotics

Probiotics are live microorganisms that, when administered in adequate quantities, confer health benefits to the host [87,88,89,90]. Current understanding suggests that their effects result primarily from direct interaction with the host and from functional modulation of the microbiota, rather than from permanent changes to bacterial composition [23].

Within pharmacomicrobiomics, several studies have shown that specific probiotic strains can alter the metabolism and bioavailability of various drugs. A formulation containing *Streptococcus salivarius* and *Lactobacillus acidophilus* increased azoreductase activity ex vivo, enhancing sulfasalazine metabolism, though without significant changes in plasma drug concentrations following single-dose administration, both in animal models and in individuals with rheumatoid arthritis [91,92]. The probiotic strain *Lacticaseibacillus casei* Zhang was shown to metabolise racecadotril into its active metabolites, S-acetylthiorphan and thiorphan, an effect that proved dependent on microbiota composition, where racecadotril degradation showed a strong positive correlation with *Bacteroidaceae* abundance and a negative correlation with *Clostridiaceae*, suggesting that these two families exert opposing effects on drug metabolism [93].

Alterations in drug absorption have also been reported. Administration of a formulation containing *Lactobacillus acidophilus*, *Lacticaseibacillus rhamnosus*, and *Bifidobacterium lactis* for three days increased gliclazide bioavailability in diabetic animal models, an effect possibly related to modulation of Mrp2 and Mrp3 transporter expression [4,27,94]. Similarly, concomitant administration of *Escherichia coli* Nissle 1917 significantly increased amiodarone bioavailability by up to 43% in experimental models, potentially through intestinal pH reduction, thereby increasing molecular ionization and its ability to cross the intestinal mucosa, or increased expression of the influx transporter OATP2B1. Given amiodarone’s narrow therapeutic index, even modest changes in its bioavailability may carry clinical significance [27,28].

Taken together, these examples illustrate the potential of probiotics to modulate pharmacological response, though their effects depend on the strain used, the host’s baseline microbiota composition, and the drug in question.

### 4.3. Faecal Microbiota Transplantation

FMT involves the transfer of intestinal microbiota from a healthy donor to a recipient, with the goal of restoring the composition and function of the intestinal microbial community [95]. Initially developed for the treatment of recurrent *Clostridioides difficile* infection, FMT has since been investigated in other conditions associated with intestinal dysbiosis [95,96,97,98].

In the context of pharmacomicrobiomics, FMT is of particular interest given its capacity to broadly reshape the metabolic potential of the gut microbiota. By restoring functional bacterial communities, it may alter the absorption, activation, or inactivation of various drugs, as well as modulate the activity of microbial enzymes involved in drug metabolism [27]. This could consequently influence the therapeutic response to drugs such as irinotecan, digoxin, tacrolimus, certain NSAIDs, and prodrugs such as racecadotril [4,27,93].

Although available results are promising, significant limitations remain regarding procedural standardisation, long-term safety, and the mechanistic understanding of its effects. Further studies are needed before FMT can be routinely applied to the personalisation of pharmacotherapy.

## 5. Future Perspectives and Challenges

### 5.1. Challenges for Clinical Translation

Pharmacomicrobiomics represents an important step towards optimising personalised pharmacotherapy by linking gut microbiome composition with individual drug responses. Translating this knowledge into routine clinical practice, however, remains substantially limited by several interconnected obstacles.

The complexity and dynamism of the gut microbiota itself constitute a fundamental barrier. Microbial composition and metabolic activity are continuously shaped by diet, lifestyle, host genetics, age, and environmental exposures, making it difficult to determine whether observed changes in the microbiota are attributable to drug treatment or to confounding variables that simultaneously modulate the microbial community [19,24]. Compounding this, the marked interindividual variability in microbiome composition means that differences in drug metabolism, efficacy, and toxicity are difficult to predict, and microbiome-based interventions that work in one individual may not translate to another [2,24].

A further limitation is the absence of standardised methodologies for sample collection, processing, sequencing, and bioinformatic analysis. This heterogeneity restricts cross-study comparisons and reduces reproducibility. Many of the mechanisms underlying host–microbiome–drug interactions also remain incompletely characterised, underscoring the need for mechanistic studies and predictive models capable of anticipating individual pharmacological responses [24,99].

Ethical and regulatory considerations add another layer of complexity. The use of microbiome data to guide clinical decisions raises concerns around data privacy, informed consent, and accountability for treatment outcomes. Equitable access to personalised therapies and the appropriate regulatory oversight of emerging interventions, such as probiotics, synbiotics, and FMT, whose long-term safety profiles are still being established, also require careful attention [24,100,101].

### 5.2. Future Directions

Future research should aim to address several unresolved questions central to the integration of pharmacomicrobiomics into precision medicine: which microbiome characteristics are the most reliable predictors of therapeutic response; how stable individual microbiome profiles are over time; how external factors such as diet and antibiotic use influence drug efficacy; and which host-related factors modulate microbiome activity in ways that alter pharmacological outcomes [24].

The identification of microbiome-derived biomarkers is particularly promising in this regard. In oncology, the presence of *Akkermansia muciniphila* has been associated with improved responses to immune checkpoint inhibitors [93]. In major depressive disorder, patients who responded more favourably to selective serotonin reuptake inhibitors tended to have microbial communities enriched in *Ruminococcus* spp., *Bifidobacterium* spp., and *Faecalibacterium* spp., alongside metabolic pathways associated with acetate production [102]. In type 2 diabetes, a higher abundance of *Blautia* spp. and enrichment of purine degradation and glutamine biosynthesis pathways before treatment initiation were associated with better metformin response, illustrating how functional biomarkers may predict therapeutic benefit more precisely than taxonomic composition alone, given the wide interindividual variability in response to this drug [103].

Integrating multi-omics approaches, namely metagenomics, transcriptomics, proteomics, and metabolomics, is expected to provide a more granular understanding of host–microbiome–drug interactions and to support the safe and effective clinical implementation of pharmacomicrobiomics [24,104].

However, before microbiome profiling can be meaningfully incorporated into routine therapeutic decision-making, candidate biomarkers will require prospective validation in adequately powered cohorts, followed by interventional studies demonstrating that microbiome-guided treatment adjustments translate into measurably improved clinical outcomes compared with standard care [105]. Only once this evidentiary standard, analogous to that required for other precision medicine biomarkers, has been met will microbiome profiling be positioned for integration into clinical guidelines and reimbursement frameworks.

## 6. Conclusions

Pharmacomicrobiomics has highlighted the gut microbiome as a central determinant of interindividual variability in drug response. Through direct and indirect mechanisms, the intestinal microbiota influences drug absorption, metabolism, efficacy, and toxicity, while pharmacological therapies can, reciprocally, alter microbial composition and function.

These bidirectional interactions offer a biological explanation for differences in therapeutic outcomes among patients receiving the same medication and reinforce the importance of accounting for the microbiome when designing treatment strategies. Approaches such as prebiotics, probiotics, and FMT have shown potential to optimise therapeutic efficacy, reduce treatment-related toxicity, and, in selected settings, restore microbiome function, though they introduce their own challenges in terms of standardisation and regulatory oversight.

Significant barriers remain, including microbiome complexity, methodological heterogeneity, and ethical considerations, but advances in biomarker identification and multi-omics technologies are progressively expanding the clinical potential of this field. Pharmacomicrobiomics complements host genomic information by incorporating the dynamic contribution of the gut microbiome to drug response. Although substantial challenges remain, continued advances in microbiome science are likely to facilitate the integration of pharmacomicrobiomics into future precision medicine approaches.

## Figures and Tables

**Table 1:** Representative examples of microbiome-mediated drug metabolism and associated clinical effects.

Drug	Microbial Mechanism	Bacterial Species/Enzymes Involved	Clinical Impact	Level of Evidence	References
Amiodarone	pH modulation (indirect)	*Escherichia coli* Nissle 1917	Increased bioavailability and toxicity risk	Animal (rat)	[28]
Amlodipine	Increased bioavailability (dysbiosis)	Not identified	Risk of accumulation and toxicity	Animal (rabbit; rat) Human	[29,30]
Clonazepam/Nitrazepam	Nitro-reduction	Nitroreductases	Formation of 7-aminoclonazepam (increased toxicity)	Animal (rat)	[31]
Cyclophosphamide	Immune modulation (indirect)	*Enterococcus hirae*, *Lactobacillus* spp.	Enhanced antitumour immune response	Animal (mouse)	[32,33]
Diclofenac	Glucuronide deconjugation	β-glucuronidases	Increased gastrointestinal toxicity	Animal (mouse)	[34,35]
Digoxin	Reduction	Cardiac glycoside reductase (*Eggerthella lenta*)	Formation of inactive dihydrodigoxin	In vitro + Animal (mouse)	[36]
Oestrogens (oral contraceptives)	Glucuronide deconjugation	β-glucuronidases (estrobolome)	Increased enterohepatic recirculation; potential reduced contraceptive efficacy with antibiotics	In vitro	[37,38,39]
Gemcitabine	Deamination/inactivation	Cytidine deaminase (*Mycoplasma hyorhinis*, *Enterobacteriaceae*)	Reduced antitumour efficacy	Animal (mouse) + human tumour samples	[40]
Indomethacin	Glucuronide deconjugation	β-glucuronidases	Increased gastrointestinal toxicity	Animal (mouse)	[34,35]
Irinotecan	Glucuronide deconjugation	β-glucuronidases	Reactivation of SN-38 (GI toxicity)	Animal (mouse)	[41]
Levodopa	Decarboxylation	Tyrosine decarboxylase (*Enterococcus faecalis*)	Reduced bioavailability	In vitro + human metagenomic association	[42]
Loperamide oxide	Reduction	Not identified	Formation of active loperamide	Animal (rat, dog) + human	[43]
Lovastatin	Ester hydrolysis	Esterases (microbial and host)	Formation of active metabolite	Animal (rat)	[44]
Metformin	Microbiome modulation	*Bacteroidota*, *Escherichia* spp., *Akkermansia muciniphila*	Improved glycaemic control (SCFA-mediated)	Observational/clinical human + Animal (mouse)	[45,46]
Metronidazole	Nitro-reduction	Nitroreductases (*Clostridium perfringens*)	Formation of active metabolite	Animal (rat, mice)	[47,48]
Midazolam	Reduced hepatic metabolism (CYP3A4)	Not identified	Increased plasma levels and toxicity	Animal (mouse)	[49]
Morphine	Glucuronide deconjugation	β-glucuronidases	Increased bioavailability and prolonged effect	Clinical human (PK) + Animal (murine)	[50,51]
Mycophenolate mofetil	Glucuronide deconjugation	β-glucuronidases	Reactivation of MPA; GI toxicity	Clinical human (kidney transplant)	[52]
Nivolumab/Pembrolizumab	Immune modulation (indirect)	*Akkermansia muciniphila*, *Faecalibacterium prausnitzii*, *Bifidobacterium* spp.	Enhanced or reduced antitumour immune response	Animal (mouse) Observational/clinical human	[53,54]
Olsalazine	Azo bond reduction	Azoreductases	Formation of active 5-ASA	In vitro + Clinical human	[55,56]
Omeprazole	Altered absorption/metabolism (dysbiosis)	Not identified	Variability in bioavailability	Animal (rat)	[57]
Paracetamol	Competition in hepatic sulfation (indirect)	Microbial metabolite p-cresol	Increased risk of hepatotoxicity	Clinical human	[58]
Prontosil	Azo bond reduction	Azoreductases	Formation of active sulfanilamide	Animal (rat), 1971—historical/foundational	[59]
Sorivudine + 5-FU	Microbial metabolism of sorivudine to a DPD inhibitor (BVU)	Not fully defined	Increased 5-FU toxicity (drug–drug interaction)	Clinical (case-based) + mechanistic	[60,61]
Sulfasalazine/Balsalazide	Azo bond reduction	Azoreductases (*Bacteroides* spp., *Clostridium* spp., *Eubacterium* spp.)	Release of active 5-ASA	In vitro (human faecal slurry) + animal (mouse, rat, ferret) toxicology/PK	[62]
Tacrolimus	Microbial metabolism	*Faecalibacterium prausnitzii, Prevotella copri*	Altered bioavailability and toxicity risk	Clinical human + in vitro	[63,64]
Warfarin	Vitamin K synthesis modulation	*Bacteroides* spp., *Lactobacillus* spp., *Escherichia* spp. *Shigella* spp., *Klebsiella* spp. and *Enterococcus* spp.	Altered anticoagulant effect; INR fluctuation with antibiotic use	Clinical human (cardiac surgery) + Animal (rats)	[24,65,66]
Daikenchuto (TU-100)	Glycoside hydrolysis	Gut bacterial glycosidases	Production of bioactive ginsenoside metabolite (compound K)	Animal (mouse) + in vitro (human colonic model)	[67,68]

Abbreviations: 5-ASA—5-aminosalicylic acid; 5-FU—5-fluorouracil; BVU—bromovinyluracil; CYP3A4—cytochrome P450 3A4; DPD—dihydropyrimidine dehydrogenase; GI—Gastrointestinal; MPA—mycophenolic acid; SCFA—short-chain fatty acids; SN-38-7-ethyl-10-hydroxycamptothecin. Note: Level of evidence refers specifically to the representative study cited for each interaction and does not imply this is the only evidence available; additional preclinical or clinical data may exist beyond the studies selected here.

**Table 2:** Examples of gut microbiota alterations associated with MS and their immunological implications [16,76].

Taxonomic Level	Alteration in MS Patients	Disease Implications
Phylum: *Bacillota*	Increase	Elevated during MS relapse
Phylum: *Bacteroidota*	Decrease	Elevated during MS relapse
Genus: *Prevotella*	Decrease	Associated with Th17 cell expansion
Genus: *Methanobrevibacter*	Increase	Promotes inflammation through recruitment of inflammatory cells
Genus: *Akkermansia*	Increase	May damage the intestinal barrier, producing a pro-inflammatory effect
Genus: *Clostridium*	Decrease	Associated with reduced SCFA production, Treg cells, and IL-10
Species: *Streptococcus mitis*/*Streptococcus oralis*	Increase	Associated with Th17 cell differentiation

## Data Availability

No new data were created or analyzed in this study. Data sharing is not applicable to this article.
